# B7-H3 overexpression in pancreatic cancer promotes tumor progression

**DOI:** 10.3892/ijmm.2012.1212

**Published:** 2012-12-13

**Authors:** XIN ZHAO, DE-CHUN LI, XIN-GUO ZHU, WEN-JUAN GAN, ZHI LI, FENG XIONG, ZI-XIANG ZHANG, GUANG-BO ZHANG, XUE-GUANG ZHANG, HUA ZHAO

**Affiliations:** 1 Departments of General Surgery, The First Affiliated Hospital of Soochow University, Suzhou, P.R. China; 2 Pathology, The First Affiliated Hospital of Soochow University, Suzhou, P.R. China; 3 Interventional Radiology, The First Affiliated Hospital of Soochow University, Suzhou, P.R. China; 4 Oncology, The First Affiliated Hospital of Soochow University, Suzhou, P.R. China; 5 Clinical Immunology, The First Affiliated Hospital of Soochow University, Suzhou, P.R. China

**Keywords:** pancreatic cancer, B7-H3, invasiveness, migration, progression, RNA interference

## Abstract

B7-H3, a member of the B7-family molecules, plays an important role in adaptive immune responses. In addition, B7-H3 is also expressed in several types of human cancers and is correlated with the poor outcome of cancer patients. However, its exact role in cancer is not known. In the present study, we compared B7-H3 expression in normal pancreas and pancreatic cancer tissue specimens, and determined the effects of low B7-H3 expression on the human pancreatic cancer cell line Patu8988 using lentivirus-mediated RNA interference. B7-H3 expression in pancreatic specimens was determined by enzyme-linked immunosorbent assay (ELISA). A Patu8988 cell line with low B7-H3 expression was established by lentivirus-mediated RNA interference to investigate the effect of B7-H3 on cell proliferation, migration and invasion *in vitro*. By establishing subcutaneous transplantation tumor and orthotopic transplantation pancreatic cancer mouse models, the effect of B7-H3 on cell proliferation, migration and invasion was studied *in vivo*. B7-H3 in tissue samples was significantly higher in the pancreatic cancer group than in the normal pancreas group (mean ± SD, 193.6±9.352 vs. 87.74±7.433 ng/g; P<0.0001). B7-H3 knockdown by RNA interference decreased cell migration and Transwell invasion up to 50% *in vitro*. No apparent impact was observed on cell proliferation *in vitro*. In the subcutaneous transplantation tumor mouse model, the tumor growth rate was reduced by the knockdown of B7-H3. In the orthotopic transplantation pancreatic cancer mouse model, the effect of inhibiting metastasis by knocking down B7-H3 was assessed in terms of the average postmortem abdominal visceral metastatic tumor weight. This demonstrated that inhibition of B7-H3 expression reduced pancreatic cancer metastasis *in vivo*. In conclusion, B7-H3 is aberrantly expressed in pancreatic cancer. In addition to modulating tumor immunity, B7-H3 may have a novel role in regulating pancreatic tumor progression.

## Introduction

Pancreatic cancer, a highly lethal disease, is always diagnosed at an advanced stage for which there is little effective treatment. It remains the fourth most common cause of cancer-related death in the Western world (1). Due to the aggressive nature of this disease, most patients with pancreatic cancer present with local invasion or distant metastasis at the time of diagnosis, and less than 20% of patients are candidates for surgery with curative intent (2). Metastatic pancreatic cancer is relatively incurable. For pancreatic cancer, the overall 5 year survival rates are reported to be below 5% (3). Thus, understanding the molecular mechanisms of pancreatic cancer progression should be helpful to develop efficient treatments for the disease. Among new approaches, gene therapy is definitely required to improve treatment results (4–7).

B7-H3, a member of the B7 immunoregulatory family, was identified in 2001 by database searches of a human dendritic cell derived cDNA library (8). Previous studies showed that B7-H3 protein can be expressed in dendritic cells, and in the liver, lung, prostate as well as in similar tumor cell lines (9–12). However, the physiological and pathological role of B7-H3 is largely unknown. In an early study, human B7-H3 was reported to be a co-stimulator of T cells, promoting T cell proliferation and cytokine production (8). Subsequently, it was reported that in several mouse cancer models, B7-H3 ectopic expression enhanced the induction of tumor-specific CD8 cytotoxic T cells, which may slow tumor growth or even completely eradicate tumors (13,14). More recently, B7-H3 was repeatedly implicated as a potent inhibitor of T cell activity (15). Previous studies found that B7-H3 deficient mice show airway inflammation (16), experimental autoimmune encephalitis (17) and allergic conjunctivitis in an accelerated pattern (18). In contrast to these studies, Steinberger *et al*(10) suggested that B7-H3 has no characteristics of a co-signaling molecule and it does not act as a regulator of immune responses. Therefore, the biological functions of B7-H3 are still unclear.

Metastasis, the spread of cancer cells from the primary tumor sites to distant organs, is a complex process that involves induction of cell motility, activation of extracellular matrix proteases, intravasation to vessels, travel via the circulatory system, and survival and establishment of secondary tumors in a new microenvironment (19,20). The same process occurs in metastatic pancreatic cancer.

It has been suggested that B7-H3 is a tumor-associated antigen that regulates important cellular responses, such as proliferation, adhesion and metastasis, indicating its novel role in tumor progression (21,22). In this study, we focused on B7-H3 in pancreatic cancer tissue as well as in the human pancreatic cancer cell line Patu8988.

## Materials and methods

### Reagents

The anti-human B7-H3 antibody was purchased from R&D Systems, Inc. The horseradish peroxidase-conjugated secondary anti-mouse antibody was from Bio-Rad Laboratories, Inc. TRIzol reagent and MMLV were purchased from Gibco-BRL. TaqDNA polymerase, dNTPs and DNA marker were purchased from Takara.

### Patients

This study was approved by the Ethics Committee of The First Affiliated Hospital of Soochow University for Clinical Investigation. Included in the study were 26 patients with pancreatic cancer who underwent surgery for radical resection. Patients were excluded from analysis if they received chemotherapy or radiation therapy prior to the surgical operation or underwent previous pancreatic surgery. Specimens of pancreatic cancer were obtained from the patients during surgical operation, following written consent. At the same time, specimens of normal pancreatic tissues distant to the tumor were obtained as controls. The diagnosis of each tissue was confirmed by a frozen section stained with hematoxylin and eosin. After dissection under sterile conditions, each tissue sample was collected, separated and divided into 2 groups during preparation and analysis. One group was fixed in 10% buffered methanol for immunohistochemical estimation of B7-H3 expression, and another group was used for B7-H3 enzyme-linked immunosorbent assay (ELISA).

### Tissue extracts and B7-H3 enzyme-linked immunosorbent assay (ELISA)

Each tissue sample was collected and weighed in the same manner during preparation. Extract preparation was performed as recommended by the manufacturer (Cell Signaling Technology). Briefly, each tissue sample was prepared with PBS and homogenized in cell lysis buffer containing Protease Inhibitor Cocktail Set I (Calbiochem). After incubation on ice for 30 min, the homogenate was centrifuged at 14,000 x g for 10 min at 4°C, and the supernatant was collected for ELISA assay. ELISA kits produced at our laboratory were used to measure B7-H3, as described previously (19). B7-H3 concentrations were determined using a standard curve with an 8-parameter curve fit analysis program. We calculated the B7-H3 level/gram (g) of each tissue sample.

### Cells and cell culture

Pancreatic cancer cell line Patu8988 was kindly provided by Professor Chang-Geng Ruan from the Jiangsu Provincial Institute of Hematology, China. Patu8988 cells were cultured in RPMI-1640 (Gibco Inc.), and the medium was supplemented with 10% fetal bovine serum (Atlanta Biologicals, Inc.) and 1% penicillin-streptomycin (Gibco, Inc.) at 37°C in an atmosphere of 5% CO_2_. After cells attained 80 to 90% confluence, they were harvested with 0.25% trypsin and split at a 1:3 ratio.

### Generation of stable cell lines

Small hairpin RNA (shRNA) of the human B7-H3 (NM_001024736; GenBank) lentiviral gene transfer vector encoding the green fluorescent protein (GFP) sequence was constructed by Shanghai GeneChem Co. (Shanghai, China). The targeting sequence of B7-H3 was 5′-GAGCAGGGCTTGTTTGATGTG-3′, and it was confirmed by sequencing. The recombinant lentivirus of small hairpin interference RNA targeting B7-H3 (LV-B7-H3 virus) and the nontargeted control mock lentivirus (LV-NC virus) were prepared and titered to 5×10^9^ Tu/ml (transfection unit). Cells were subcultured at 5×10^4^ cells/well into 6-well tissue culture plates overnight. The viral supernatant was then added into cells at a multiplicity of infection (MOI) of 10 with ENi.S and 5 μg/ml Polybrene. GFP was evaluated by fluorescence microscopy to estimate the infection efficiency. The infected Patu8988 cells were termed the LV-B7-H3 group and LV-NC group, respectively, and the Patu8988 cells without infection were the control group. The 3 groups mentioned above were used in subsequent experiments. Real-time reverse transcriptase-polymerase chain reaction (RT-PCR) was carried out to confirm the knockdown of B7-H3 mRNA, and B7-H3 protein expression was analyzed by FCM using a Cytomics™ FC 500 device.

### Real-time reverse transcriptase-polymerase chain reaction (RT-PCR)

RT-PCR was performed to confirm the knockdown of B7-H3 mRNA in the transfectants. Total RNA was collected using TRIzol reagent following the manufacturer’s instructions. The concentration and purity of the total RNA were detected with an ultraviolet spectrophotometer and then reversely transcribed into cDNA with MMLV. Quantitative real-time PCR assays were carried out using SYBR-Green Real-time PCR Master Mix and real-time PCR amplification equipment. GAPDH was used as an internal control. The PCR conditions consisted of 1 cycle at 95°C for 15 sec followed by 45 cycles at 95°C for 5 sec and at 60°C for 30 sec. The primer sequences were as follows: 5′-CTCTGCCTTCTCACCTCTTTG-3′ (sense) and 5′-CCTTGAGGGAGGAACTTTATC-3′ (antisense) for B7-H3 (134 bp); 5′-TGACTTCAACAGCGACACCCA-3′ (sense) and 5′-CACCCTGTTGCTGTAGCCAAA-3′ (antisense) for GAPDH (121 bp).

In another experiment of RT-PCR for B7-H3, products were electrophoresed on 1.8% agarose gel containing 0.1% ethidium bromide. Images of the fluorescent bands were captured by use of the Bio-Rad gel documentation system.

### Cell proliferation by MTT assay

The MTT assay was used to study the effect of B7-H3 RNA interference on Patu8988 cell proliferation. Cells of each group were plated at 10,000 cells/ well in a 96-well plate for 24, 48 or 72 h. At each time point after discarding the medium, 100 μl RPMI-1640 containing 20 μl MTT (Sigma) (5 mg/ml) was added to each well. After incubation at 37°C for 4 h the MTT solution was removed. Dimethyl sulfoxide (100 μl) was added to each well and mixed to dissolve the dark blue formazan crystals that formed. The proportion of viable cells was determined by reading the optical density using test wave length (570 nm) and reference wave length (630 nm) with a Multiskan™ MK3 ELISA reader. The assay was carried out in quintuplicate for each group and repeated in triplicate.

### In vitro wound scrape assay

Cells of each group were incubated in 6-well plates. A small wound area was made in the confluent monolayer with a 200-μl pipette tip in a lengthwise stripe. Cells were then washed twice with PBS and incubated in serum-free RPMI-1640 medium at 37°C in a 5% CO_2_ incubator for 24 h (23,24). Images were captured at different times from 0 to 48 h. Wound width was measured at a ×100 magnification using a BX50 microscope (Olympus) with a calibrated eyepiece grid (1 mm/100 μm graduation). Ten measurements were determined at random intervals along the wound length. This experiment was carried out in triplicate.

### In vitro invasion assay

A co-culture system was used as an alternative method to evaluate cancer cell invasiveness (25). Briefly, the upper portion of Transwell inserts with an 8-μm pore size and a 6.5-mm diameter was coated with 20 μl Matrigel diluted 1:3 in serum-free RPMI-1640 and incubated at 37°C for 4 h. The coated inserts were placed in the well of a 24-well plate with 600 μl RPMI-1640 containing 10% FBS in the bottom chamber. After 12 h of serum starvation, the trypsinized cells were harvested and diluted to a 5×10^6^/ml cell suspension with serum-free RPMI-1640. Each cell suspension (100 μl) was added to the upper chambers. After incubation at 37°C for 48 h in a 5% CO_2_ atmosphere, the non-invading cells and gel were removed from the upper chamber with cotton tipped swabs. The cells were rinsed with PBS, and cells on the filters were fixed with methanol for 30 min and stained with crystal violet solution (Sigma). The number of invading cells on the filters was counted in 5 random fields/filter at ×100 magnification in triplicate wells of each group.

### Subcutaneous transplantation model study

Three groups of 6 male Balb/c nude mice (5- to 6-weeks old and 20–24 g in weight) were bred in an aseptic-specified pathogen-free (SPF) condition and kept at a constant humidity and temperature (25–28°C). Animal experiments were carried out according to protocols approved by the Animal Care and Use Committee and were in compliance with the Guidelines on Animal Welfare of the China National Committee for Animal Experiments. Cells (2×10^7^) (LV-B7-H3, LV-NC or control cells) in 0.2 ml normal sodium were injected subcutaneously in the right inguinal region of nude mice, respectively. The size of tumors was measured twice a week with calipers, and the volume was determined using the simplified formula of a rotational ellipsoid (L x W^2^ × 0.5). Growth curves were constructed, and the data are presented as means ± SD. Tumors were harvested from mice 6 weeks after tumor cell injection. B7-H3 expession was detected by immunohistochemistry of the tumor xenografts.

### Immunohistochemistry

Clinical specimens and the tumor xenografts were used for immunohistochemical studies. Specimens were fixed in formalin overnight and embedded in paraffin. Serial sections (4 μm) were prepared for immunohistological staining. Tissue sections were quenched for endogenous peroxidase with freshly prepared 3% H_2_O_2_ with 0.1% sodium azide and then placed in an antigen retrieval solution for 15 min. After incubation in a casein block, primary antibodies such as anti-B7-H3 (1:50 dilution) were applied to the sections for 1 h at room temperature, followed by incubation with the secondary antibody and Extravidinconjugated horseradish peroxidase. The immune reaction was counterstained with hematoxylin, dehydrated, and mounted. Sections were then evaluated for the presence of brown diaminobenzidine precipitates indicative of positive reactivity by microscopy. The brown staining in the cytoplasm was read as positive reactivity for B7-H3.

### Orthotopic transplantation pancreatic cancer model study

The establishment of an inguinal region subcutaneous transplantation tumor model of 3 groups (LV-B7-H3, LV-NC and control cells) was carried out respectively, as described above. After growing to a specific certain volume, the tumors were resected under aseptic environment and washed twice in antibiotic-containing RPMI-1640 to prevent possible infection. Necrotic tissues were removed, and the remaining viable tumor tissues were cut into small pieces of 1 mm^3^. Five-week-old BALB/c-nu mice, weighing 20–24 g, were anesthetized with urethane (4 ml/kg) by intramuscular injection. After the abdominal skin was sterilized, an incision was made in the upper left abdomen, and the pancreas was exposed. Tumor pieces were attached to the pancreas using absorbable sutures. The pancreas was then returned to the peritoneum, and the abdominal wall and the skin were closed with silk sutures. The animals were allowed to recover for 24 h. Three groups of 6 surviving mice were bred in an aseptic-specified pathogens-free (SPF) condition and kept at a constant humidity and temperature (25–28°C). All of the mice were sacrificed 7 weeks after the orthotopic transplantation operation. Metastatic visceral tumors out of the pancreas, such as metastatic tumors in liver, on the small intestine serous membrane surface or on the peritoneum, were excised carefully and weighed as described previously (26).

### Statistical analysis

B7-H3 expression in pancreatic cancer and normal pancreas tissues as determined by immunohistochemical staining was compared and assessed using the Chi-square test. Other data are shown as means ± SD. Statistical comparisons were performed using the Student’s t-test. All P-values were determined by 2-sided tests with significance considered at <0.05. These analyses were performed using SPSS 13.0 software.

## Results

### Tissue samples and immunohistochemical staining

**Figure 1:**
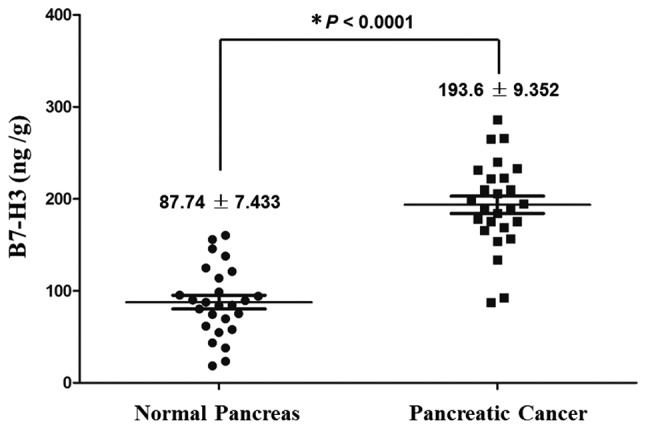
B7-H3 was significantly higher in pancreatic cancer than in normal pancreas tissue samples. (^*^P<0.0001, pancreatic cancer vs. normal pancreas).

**Figure 2:**
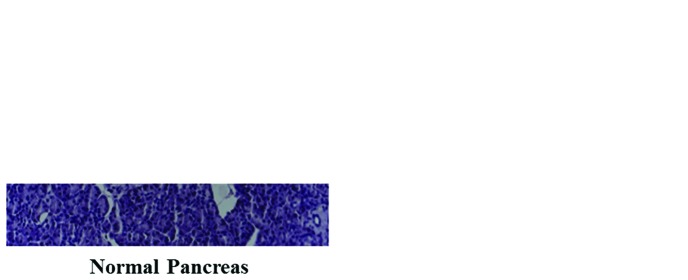
Immunohistochemical staining for B7-H3 in clinical specimens. (A) Low expression in normal pancreas. (B) Overexpression in pancreatic cancer tissue. (Magnification, ×200).

B7-H3 levels in the pancreatic cancer group were significantly higher than that in the normal pancreas group (mean 193.6±9.352 vs. 87.74±7.433 ng/g, P<0.0001) (Fig. 1). Immunohistochemical staining revealed significantly overexpressed B7-H3 in tumor tissue (Chi-square test 15.341; P<0.001). Positive staining for B7-H3 expression was detected in more than 50% of cells in 17 of the 26 pancreatic cancer specimens while no positive cells were detected in normal pancreas specimens (Fig. 2).

### B7-H3 downregulation by RNA interference in Patu8988 cells

**Figure 3:**
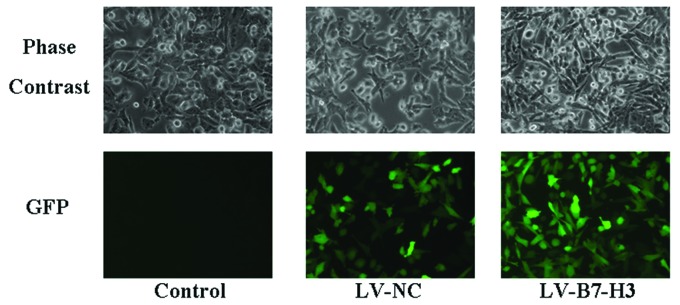
Efficiency of infection as detected by GFP expression using fluorescence microscopy. Patu8988 cells were infected with lentivirus LV-B7-H3 and lentivirus LV-NC, respectively. Phase contrast and GFP expression were assessed under a fluorescence microscope. (Magnification, ×200).

**Figure 4:**
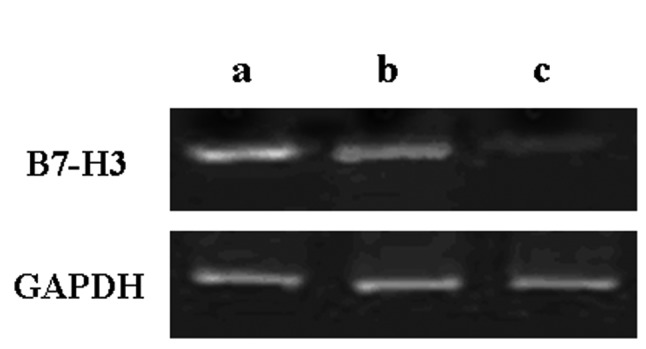

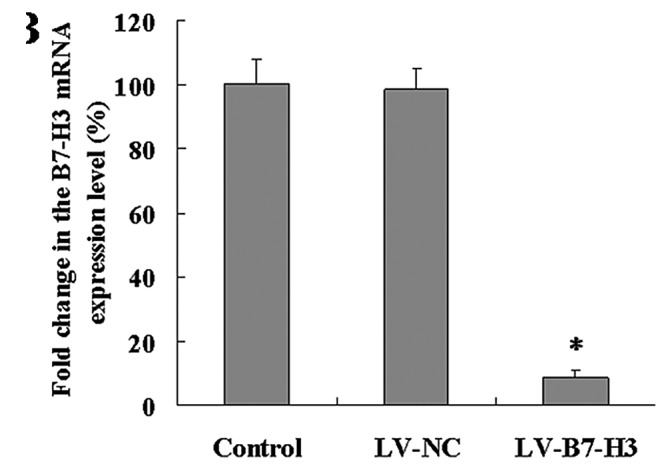

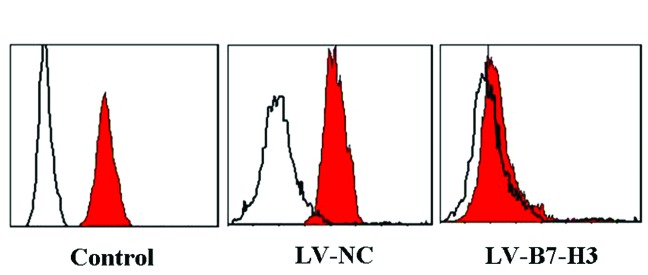
Silencing effect of B7-H3. (A) RT-PCR of B7-H3 mRNA in the 3 groups was performed with GAPDH as the loading control (lane a, control; lane b, LV-NC; lane c, LV-B7-H3). B7-H3 mRNA in the LV-B7-H3 group was knocked down vs. the other 2 groups. (B) The knockdown effect of B7-H3 mRNA by real-time RT-PCR. Relative expression of B7-H3 mRNA level was analyzed using the 2^−ΔΔCt^ method. B7-H3 mRNA expression was significantly inhibited in the LV-B7-H3 group (^*^P<0.01 vs. the control group). (C) The knockdown effect of B7-H3 protein by FCM. The protein expression level of B7-H3 in the LV-B7-H3 group was obviously downregulated vs. that of the other 2 groups (P<0.01).

After infection with the lentiviral vector, Patu8988 cells were examined by fluorescence microscopy (Fig. 3). The result showed high efficiency of the lentiviral infection. To determine the efficiency of RNA interference, we analyzed the levels of B7-H3 mRNA and protein expression in the 3 groups. Fig. 4A and B shows B7-H3 mRNA expression in the 3 groups. B7-H3 mRNA expression was obviously decreased in the LV-B7-H3 group compared with the LV-NC or the control groups (mean 8.4±2.15%, P<0.01) (Fig. 4B). The inhibition rate was 91.6%. However, there was no significant difference between the LV-NC group and the control group (P>0.05). A similar decrease was found in protein synthesis by FCM assay (mean 20.6±5.9%, P<0.01) (Fig. 4C). The mean inhibition rate was 79.4% vs. the control group. These findings indicate that the downregulation of the B7-H3 gene, by RNA interference was specific and efficient.

### Proliferation in vitro by MTT assay

**Figure 5:**
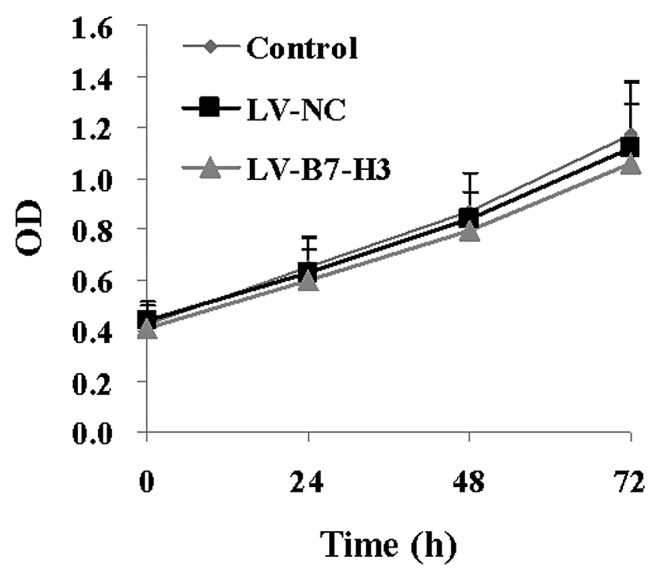
Cell proliferation as assessed by MTT. Data are expressed as the means ± SD of 3 independent experiments in triplicates (OD, optical density). Curves of cell growth after infection for 24, 48 and 72 h were established by MTT assay. There was no statistically significance difference in cell proliferation between the LV-B7-H3 and the control group (P>0.05).

To characterize the role of B7-H3 in Patu8988 cell growth, we measured the cell proliferation rate *in vitro* by MTT assay. There was no statistical significance in cellular proliferation between the control and the experimental groups (P>0.05) (Fig. 5).

### Migration on wound scrape assay in vitro

**Figure 6:**
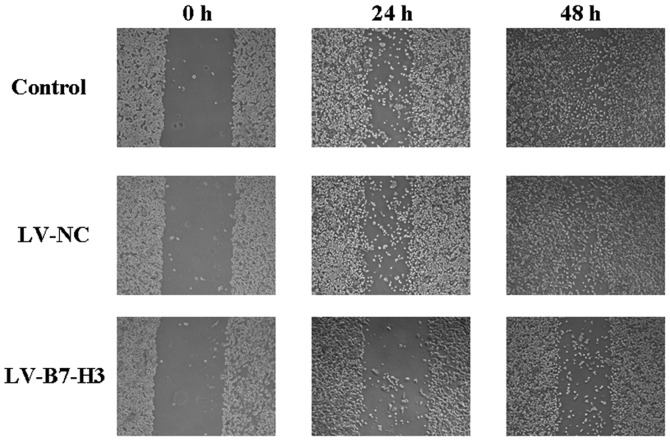
Cell migration as detected by wound scrape assay *in vitro*. Cells were damaged by mechanical scraping. Representative monolayer images of cell migration in the wound scrape model at 0, 24 and 48 h are shown. (Magnification, ×200).

To determine whether B7-H3 acts as a cell migration regulator, we used the wound scrape assay to evaluate cell motility. RNA interference resulting in inhibition of B7-H3 significantly decreased Patu8988 cell migration in the wound scrape model (Fig. 6). Time course analysis of the wound closure showed that a monolayer was re-established within a significantly shorter period in the LV-NC and control groups than that in the LV-B7-H3 group.

### Invasive ability in the Transwell assay in vitro

**Figure 7:**
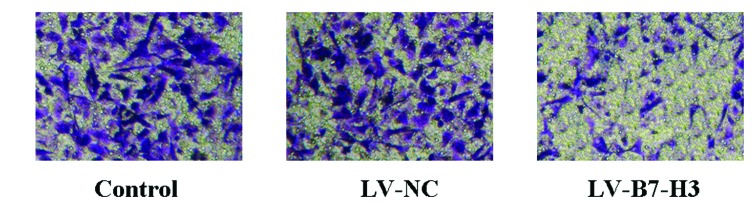

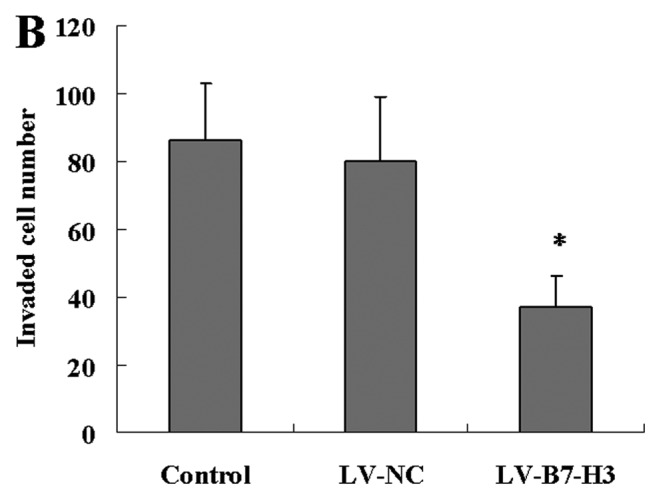
Cell invasive ability as detected by Transwell assay. (A) Representative images of invading cells. (Magnification, ×100). (B) The number of invading cells are expressed as the mean ± SD of 3 independent experiments. (^*^P<0.05, LV-B7-H3 group vs. the control group).

After down-regulation of the expression of B7-H3 by RNA interference, an *in vitro* assay on Matrigel filters revealing that the number of invading Patu8988 cells was decreased up to 50% (P<0.05, LV-B7-H3 group vs. the control group) (Fig. 7). There was no statistical significance in the number of invading cells between the LV-NC and the control group (P>0.05).

### Tumor growth in the subcutaneous transplantation mouse model

**Figure 8:**
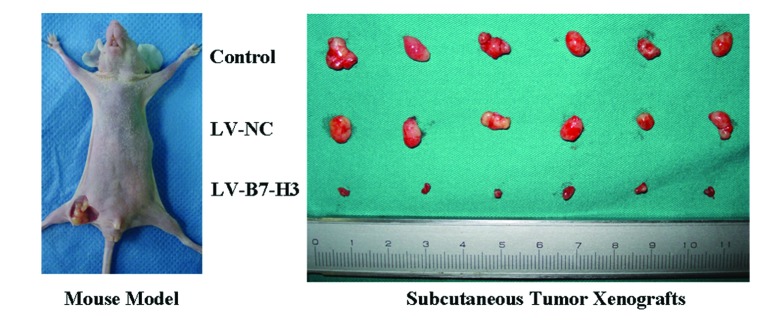

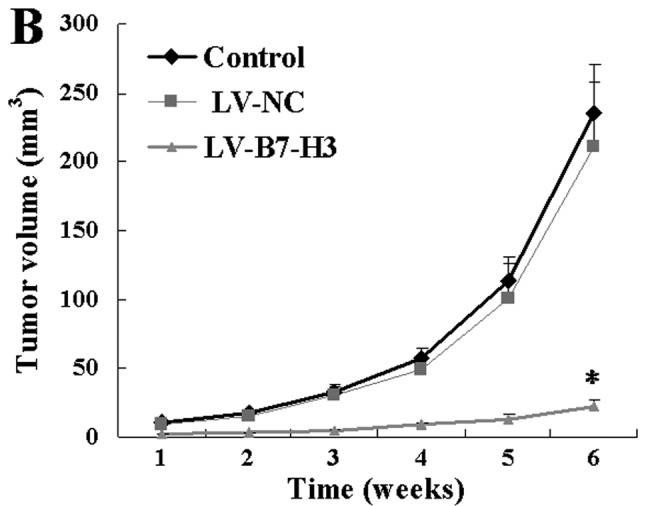
Tumor growth in the subcutaneous transplantation mouse model. (A) A subcutaneous transplantation mouse model was established, and subcutaneous transplantation xenografts were excised after 6 weeks. (B) Growth curves of pancreatic cancer subcutaneous xenografts in nude mice are shown. Each group consisted of 6 mice, and the data are expressed as means ± SD. The tumor volume of the LV-B7-H3 group was obviously smaller (^*^P<0.01, vs. the control group).

The *in vitro* experiments with the Patu8988 cells showed the effects of B7-H3 on tumor progression. Hence, we examined whether this could be observed *in vivo*. LV-B7-H3, LV-NC and control cells were injected subcutaneously into nude mice. All of the 18 mice developed detectable tumors at the beginning of this experiment. The growth rate was reduced by the knockdown of B7-H3 (Fig. 8). Inhibition of tumor growth was observed in the LV-B7-H3 group at 6 weeks, when compared to the LV-NC group (211±47 mm^3^) or the control group (235±57 mm^3^). The average tumor volume (22±5 mm^3^) in the LV-B7-H3 group was significantly lower than that in the LV-NC and control groups (P<0.01). There was no statistical significance in tumor volume between the LV-NC and the control group (P>0.05).

**Figure 9:**
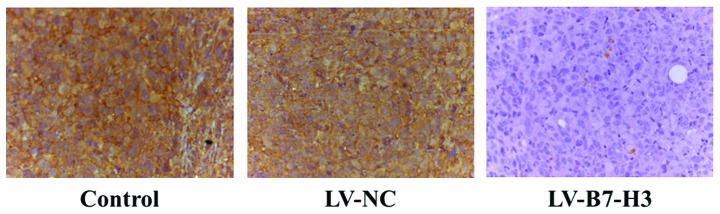
Immunohistochemical staining for B7-H3 in subcutaneous xenotransplantation tumors. Xenografts from the LV-NC and the control group showed distinct membranous and cytoplasmic immunoreactivity for B7-H3, while xenografts from the LV-B7-H3 group showed weak B7-H3 expression. (Magnification, ×400).

The knockdown of B7-H3 in the xenografts was confirmed by immunohistochemical staining. While the level of B7-H3 expression retaimed low in the LV-B7-H3 group tumors, the LV-NC and control tumors showed strong staining (Fig. 9).

### Metastatic tumors in the orthotopic transplantation pancreatic cancer mouse model

**Figure 10:**
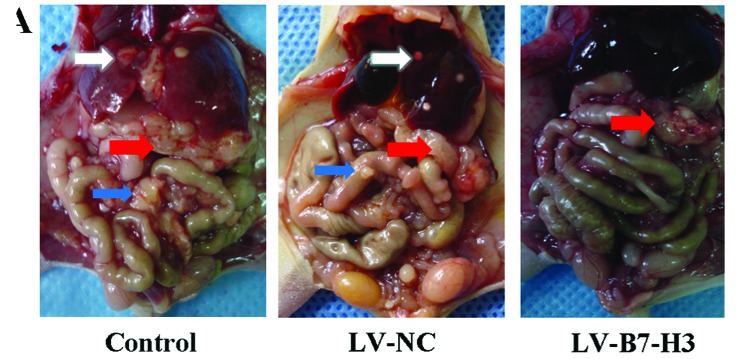

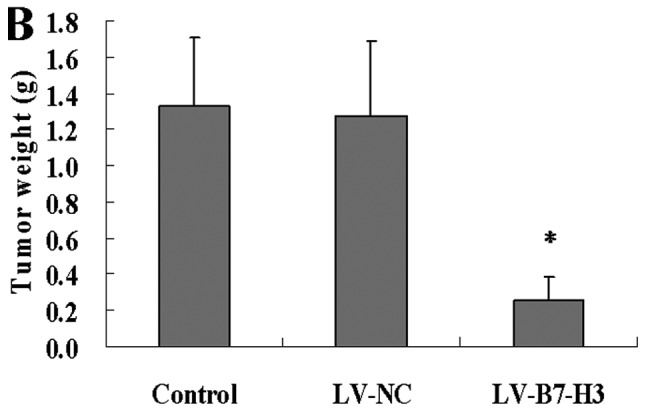
Metastatic tumors in the orthotopic transplantation pancreatic cancer mouse model. (A) An orthotopic transplantation pancreatic cancer mouse model was established. Orthotopic pancreatic cancer tumors, liver metastatic tumors and abdominal cavity metastatic tumors are indicated by red, white and blue arrows, respectively. (B) Seven weeks after the orthotopic transplantation operation, metastatic visceral tumors out of the pancreas were excised and weighed. Each group consisted of 6 animals, and the data are presented as means ± SD. The tumor weight of the LV-B7-H3 group was obviously lower when compared with the LV-NC and control group (^*^P<0.01 vs. the control group).

All of the 18 mice were sacrificed 7 weeks after the transplantation operation. All of the mice developed orthotopic transplantation pancreatic cancer tumors in this experiment. Abdominal visceral metastatic tumors were detected, excised and weighed (Fig. 10). The number of cases of liver metastasis in the LV-B7-H3 group (1/6, 16.67%) was less than the number of cases in the LV-NC group (4/6, 66.67%) or the control group (5/6, 83.33%). The effect of inhibiting metastasis by knockdown of B7-H3 was assessed in terms of the average postmortem abdominal visceral metastatic tumor weight. Inhibition of metastasis was observed in the LV-B7-H3 group, when compared to the LV-NC group (1.28±0.41 g) or the control group (1.33±0.38 g). The average weight of the abdominal visceral metastatic tumors (0.26±0.13 g) in the LV-B7-H3 group was significantly lower than that in LV-NC and control groups (Fig. 10B) (P<0.01). There was no statistical significance in metastatic visceral tumor weight between the LV-NC and the control group (P>0.05). These indicated that inhibition of B7-H3 expression reduced pancreatic cancer metastasis *in vivo*. It strongly supports the effects observed *in vitro* indicating that B7-H3 plays a vital role in invasion and migration of pancreatic cancer cells.

## Discussion

In recent years increasing evidence indicates that B7-H3 plays an important role in tumor progression and metastasis. Wu *et al*(27) reported that B7-H3 expression is related to survival time and tumor infiltration depth in gastric cancer cases. Zhang *et al*(28) found that circulating B7-H3 in serum is a highly sensitive biomarker for non-small cell lung cancer (NSCLC) and increased circulating B7-H3 suggests a poor clinical prognosis for NSCLC patients. Sun *et al*(29) reported that higher B7-H3 expression in colorectal cancer was positively correlated with a more advanced tumor grade, and the level of soluble B7-H3 in serum from colorectal cancer patients was higher than healthy donors. This suggests that both soluble and membranous B7-H3 proteins are involved in colorectal cancer progression. Yamato *et al*(12) found that B7-H3 expression was significantly more intense in cases with lymph node metastasis and advanced pathological stage in pancreatic cancer. B7-H3 blockade induced a substantial antitumor effect on murine pancreatic cancer. B7-H3 overexpression was also reported to correlate with tumor aggressiveness and poor clinical outcome, suggesting that B7-H3 has a critical role in tumor progression.

Since we realized the limitation of using immunohistochemical methods for semi-quantitative analysis (30), we also used ELISA. Our results showed aberrant B7-H3 expression in pancreatic cancer, in accord with the findings of Yamato *et al*(12). However, to ascertain why B7-H3 overexpression correlates with pathological indicators of aggressive cancer and clinical outcome and its role in tumor progression, we further investigated the effects of low B7-H3 expression on the biological features of human pancreatic cancer Patu8988 cells.

Carcinogenesis is a multiple step process in which cancer cells lose proliferation control, disseminate from a localized primary tumor mass to invading adnexa and metastasize to distant organs. Limitless cell growth is an important alteration in cancer cell phenotype (31). We first determined the effects of B7-H3 depletion on Patu8988 cell growth. The proliferation of B7-H3-knockdown cells was the same as that of controls and there was no obvious difference. To further investigate whether B7-H3 contributes to tumor metastasis, we performed a wound scrape assay to evaluate cell motility and a Transwell invasion assay to assess cell invasiveness *in vitro* to determine the mechanisms of cell metastasis toward distant tissue. The results indicated that B7-H3 has a putatively important role in tumor migration and invasiveness, indicating higher aggressiveness and poor clinical outcome.

The results *in vitro* were confirmed in our studies *in vivo*. In the subcutaneous transplantation model, the growth rate of established B7-H3-knockdown xenografts was slower than that of the LV-NC and the control groups. Although the MTT assay *in vitro* showed that the proliferation rate was not decreased by B7-H3 knockdown, the growth rate of B7-H3-knockdown tumors in the subcutaneous transplantation model showed a significant decrease. Nevertheless, the tumor cell growth microenvironment *in vivo* is quite more complicated than that *in vitro*. The underlying molecular mechanisms of this phenomenon still require further research. Immunohistochemical analysis of the xenograft tissue confirmed that the tumors originating from LV-B7-H3 cells retained low expression levels of the B7-H3 protein, whereas the LV-NC group and the control group tumors showed strong B7-H3 staining. Furthermore, in the orthotopic transplantation pancreatic cancer model, we found that decreased B7-H3 expression reduced tumor metastasis. Compared to the control group, there was a dramatic reduction in the weight of the abdominal visceral metastatic tumors in the LV-B7-H3 group. The underlying reasons may at least partly be that B7-H3 knockdown inhibits pancreatic cancer migratory and invasive ability.

In summary, our study investigating the role of B7-H3 in pancreatic cancer progression shows that this protein promotes cancer cell migration and invasiveness *in vitro* and *in vivo*. Furthermore, in contrast to previous reports focusing on the immunoregulatory effects of B7-H3, which are involved in evasion of cancer immune surveillance, our data show that it plays a critical role in pancreatic cancer progression through cell migration and invasiveness via non-immunomechanisms. These findings provide new insight into the role of B7-H3 in pancreatic cancer and may have important implications in the development of targeted therapeutics for this disease. However, whether B7-H3 regulates cancer progression directly or through various important intracellular pathways, still requires investigation, and we will engage in this field further.

In conclusion, B7-H3 is aberrantly expressed in pancreatic cancer. Our study indicates that B7-H3 may regulate tumor progression by promoting cell migration and invasiveness, in addition to acting as an immunoregulatory protein. B7-H3 may serve as a potential molecular target for pancreatic cancer therapy. Although preliminary data are significant, a precise mechanism of B7-H3 expression regulation in the tumor environment, overall knowledge of its clinical implications and targeted therapeutic interventions in pancreatic cancer require further investigation.
